# Real-Time and Retrospective Health-Analytics-as-a-Service: A Novel Framework

**DOI:** 10.2196/medinform.4640

**Published:** 2015-11-18

**Authors:** Hamzeh Khazaei, Carolyn McGregor, J Mikael Eklund, Khalil El-Khatib

**Affiliations:** ^1^ IBM Canada Research and Development Center Markham, Toronto, ON Canada; ^2^ University of Ontario Institute of Technology Faculty of Business and IT Oshawa, ON Canada; ^3^ University of Ontario Institute of Technology Department of Electrical, Computer and Software Engineering Oshawa, ON Canada

**Keywords:** premature babies, physiological data, decision support system, analytics-as-a-service, cloud computing, big data, health informatics, real-time analytics, retrospective analysis, performance modeling

## Abstract

**Background:**

Analytics-as-a-service (AaaS) is one of the latest provisions emerging from the cloud services family. Utilizing this paradigm of computing in health informatics will beneﬁt patients, care providers, and governments signiﬁcantly. This work is a novel approach to realize health analytics as services in critical care units in particular.

**Objective:**

To design, implement, evaluate, and deploy an extendable big-data compatible framework for health-analytics-as-a-service that offers both real-time and retrospective analysis.

**Methods:**

We present a novel framework that can realize health data analytics-as-a-service. The framework is flexible and conﬁgurable for different scenarios by utilizing the latest technologies and best practices for data acquisition, transformation, storage, analytics, knowledge extraction, and visualization. We have instantiated the proposed method, through the Artemis project, that is, a customization of the framework for live monitoring and retrospective research on premature babies and ill term infants in neonatal intensive care units (NICUs).

**Results:**

We demonstrated the proposed framework in this paper for monitoring NICUs and refer to it as the Artemis-In-Cloud (Artemis-IC) project. A pilot of Artemis has been deployed in the SickKids hospital NICU. By infusing the output of this pilot set up to an analytical model, we predict important performance measures for the ﬁnal deployment of Artemis-IC. This process can be carried out for other hospitals following the same steps with minimal effort. SickKids’ NICU has 36 beds and can classify the patients generally into 5 different types including surgical and premature babies. The arrival rate is estimated as 4.5 patients per day, and the average length of stay was calculated as 16 days. Mean number of medical monitoring algorithms per patient is 9, which renders 311 live algorithms for the whole NICU running on the framework. The memory and computation power required for Artemis-IC to handle the SickKids NICU will be 32 GB and 16 CPU cores, respectively. The required amount of storage was estimated as 8.6 TB per year. There will always be 34.9 patients in SickKids NICU on average. Currently, 46% of patients cannot get admitted to SickKids NICU due to lack of resources. By increasing the capacity to 90 beds, all patients can be accommodated. For such a provisioning, Artemis-IC will need 16 TB of storage per year, 55 GB of memory, and 28 CPU cores.

**Conclusions:**

Our contributions in this work relate to a cloud architecture for the analysis of physiological data for clinical decisions support for tertiary care use. We demonstrate how to size the equipment needed in the cloud for that architecture based on a very realistic assessment of the patient characteristics and the associated clinical decision support algorithms that would be required to run for those patients. We show the principle of how this could be performed and furthermore that it can be replicated for any critical care setting within a tertiary institution.

## Introduction

Over the past few decades, our society has transitioned to a state where bottlenecks have shifted from a lack of data to limitations in extracting meaningful knowledge from an abundance of data and subsequently using that knowledge to drive decisions. This data-rich, knowledge-poor oxymoron is particularly true in computationally driven clinical decision support systems (CDSSs), where advances in automated high-throughput data acquisition and electronic health records (EHRs) have yet to be translated into knowledge extraction [1].

Adoption of EHRs and systematic collection of physiological data by health care providers were predicted to vastly improve the efficiency and quality of patient care [2]. Unfortunately, despite advances in data collection and storage, these gains have yet to be realized [3,4]. One reason for this failure is that our power to utilize complex, large-scale datasets to generate knowledge and inform clinical decisions remains limited. For example, while CDSSs have existed for decades, they are mostly limited to local alert systems and (data-oblivious) agent-based suggestions that rely on hard-coded criteria.

Recently, enabled by cloud computing Web services, advanced analytics methods have been applied and utilized across a wide spectrum of health care settings for many purposes. Cloud computing has special features for clients (eg, radiologists, physicians, researchers, and patients), aiming to reduce the burden of heavy investments and to utilize resource outsourcing, software, hardware, automated resource management, parallel computing, virtualization, and utility computing [5]. The objectives of such usage include improving patient care, augmenting less-sophisticated rules-based systems, analyzing continuous feeds of physiological data, optimizing financial processes, and resource utilization [6].

Health analytics offers many different methods for the potential improvement of patient care [7]. For example, one predictive risk assessment platform involves using risk assessment analytics to process EHR data to identify patients at the greatest risk for utilizing more resources than their peers with the goal of improving patient outcomes and managing costs. The EHR data were input into a common data model that was then processed by various analytic techniques to stratify patients as “high risk” [8]. Another method described in the literature focused on the potential value of aggregating data enhanced with real-time analytics to provide point-of-care information to oncologists that was tailored to individual patients [9]. One group reported the application of predictive analytics for better targeting of disease management and innovative patient care approaches, while also warning of the unintended consequences that may arise such as excluding disadvantaged populations [10]. Unlabeled and free-text databases such as mammography data can be transformed into computationally accessible collections that are usable for large-scale health analytics [11,12]. Analytics can supplement real-time analysis of physiological data streams in the neonatal intensive care unit (ICU) for earlier detection of worsening medical conditions [13].

Analytics is also utilized in health care applications outside of the traditional inpatient and outpatient patient care settings, such as wearable monitors that patients use at home. Wearable health monitoring systems consist of a variety of sensors, actuators, and multimedia devices, and enable low-cost, noninvasive options for continuous monitoring of health, activity, mobility, and mental status, both indoors and outdoors [14]. Thus, wearable monitoring systems provide continuous physiological data that may reflect the general health of the monitored individuals. The use of wearable sensors in health monitoring systems is an emerging health care field that necessitates data mining and analytics of physiological measurements in a nonclinical setting [15]. Such health monitoring systems may reduce health care costs by disease prevention and enhance the quality of life with disease management and can be tailored to specific uses such as intelligent health monitoring of the elderly individuals in nursing homes and for individuals with dementia or Parkinson’s disease [16,17].

These rich sources of data along with aforementioned analytics capabilities have potential for an increased understanding of disease mechanisms and better health care; however, the volume, velocity, variety, veracity, and value of medical data (ie, big data characteristics) present many challenges that limit the effectiveness of outcome for all stakeholders [8]. One promising solution that addresses all these barriers is the Health-Analytics-as-a-Service (HAaaS) paradigm. Analytics-as-a-service (AaaS), in general, is a new “as-a-service,” and it is more than just simplifying access to technology. AaaS combines the on-demand aspects of cloud computing with the democratization of information enabled by big data analytics.

In this paper, we present and evaluate a cloud-based reference framework for providing HAaaS for both real-time and retrospective analysis. The framework has the capability to provide all 4 types of analytics, that is, descriptive, predictive, prescriptive, and discovery [18], in a service-oriented fashion. It leverages the latest technologies and best practices for big data analytics and also utilizes the security and privacy measures appropriate for health and medical data. The architecture has been realized (or customized) for neonatal intensive care units (NICUs) at The Hospital for Sick Children (SickKids Hospital) in Toronto and is known as the Artemis project. We have also developed an analytical model for evaluating the performance and availability of an Artemis-IC platform in preparation for migrating Artemis to Artemis-IC. We discuss the important aspects of the system performance and capacity planning process. The main functionalities of the framework are presented via one of our developed algorithms (ie, Sepsis disease detection). We also present a high-level security and privacy schema for the framework that can be customized and extended for different health applications and use cases. We show the principle of how this could be performed and show that it can be replicated for any critical care setting within a tertiary institution that has critical care.

## Methods

In this section, we highlight the functional and nonfunctional characteristics of the framework. Two editions of the framework, research and clinical editions, are designed in such a way that support acquisition and storage of physiological data as well as clinical information, for example, EHR, for the purpose of real-time/retrospective analytics and visualization. The framework is capable of gathering physiological data from a vast variety of medical devices and transfers them in a secure way toward the back-end system residing on the cloud. However, anonymization and potential translation are in order before data leave the hospitals.

The framework has an interface for communication with each hospital’s clinical information management system to obtain complementary information (eg, admission information, laboratory test results) of patients. The framework utilizes a hospital interface, which facilitates the management of hospitals’ connectivity in various geographic locations. A hospital interface can also be used for “extract, transform, and load” (ETL) purposes as well as load balancing.

Even though the research edition is for retrospective analysis and historic data visualization, it is capable of medical rule deployment and real-time analytics. This is only for testing the new and modified medical rules before undergoing further assessment and auditing. By contrast, the clinical edition was specifically designed for real-time monitoring/visualization, and here human domain experts deploy new or modified medical rules after being extensively validated and certified.

### Research Edition

**Figure 1:**
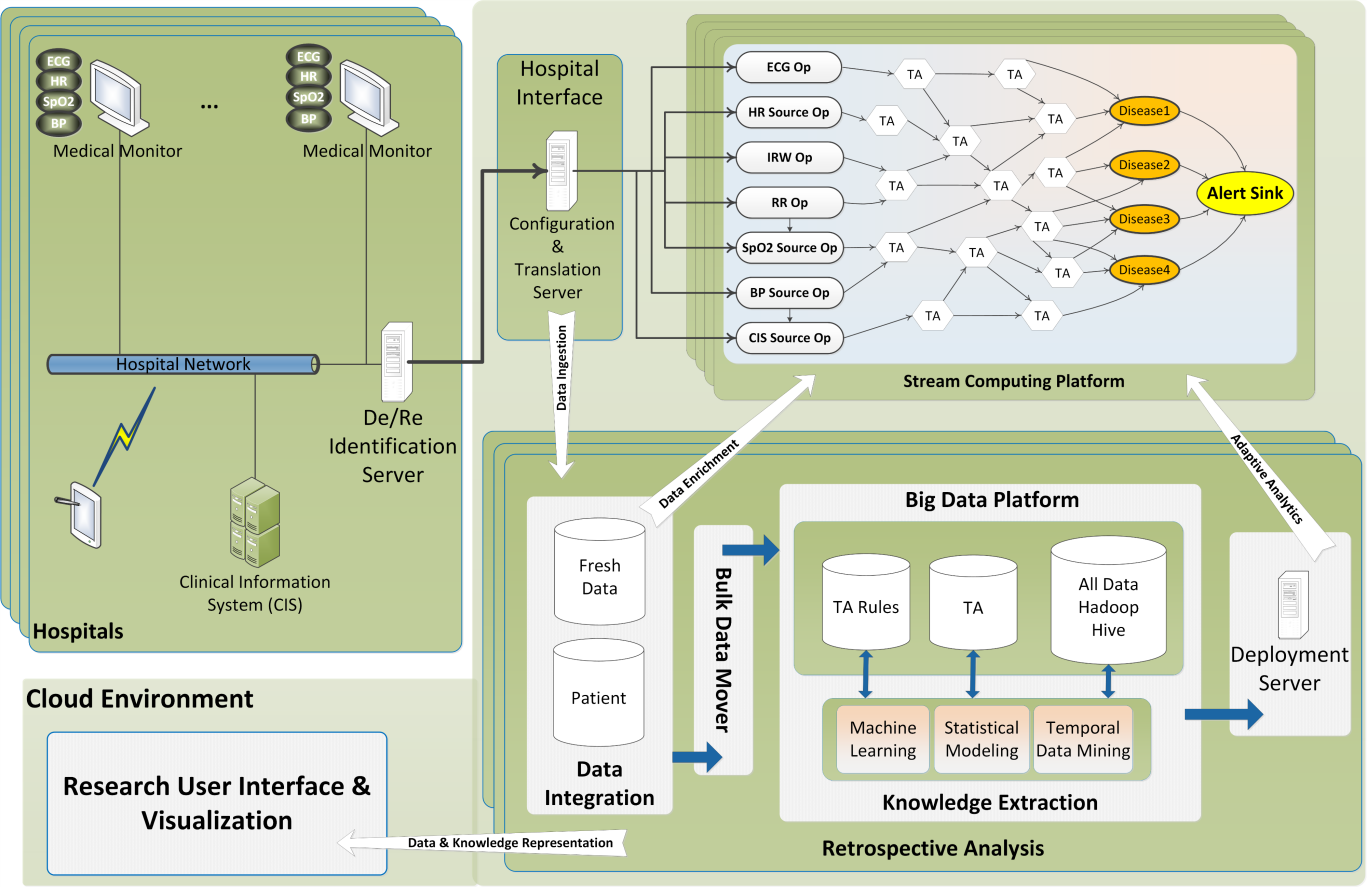
General architecture of the framework (research edition).

Researchers are the main users of the research edition (RE). This edition can be considered as a comprehensive solution that facilitates retrospective analysis on large numbers of patient data from different places. In addition to real-time analytics capabilities, the RE is able to provide at-rest analytics for stored data. Incorporating a big data analytics solution, that is, Apache Hadoop, offers great power of analysis as well as persistent storage. More specifically, the RE provides clean and ready-to-process medical data (ie, physiological, medical, laboratory, and other complementary data) along with the tools from the Hadoop ecosystem for the researchers to perform their analytics much easier than in the past. Researchers may apply knowledge discovery techniques, for example, temporal data mining [13], machine learning, and statistical modeling, against vast amounts of stored data and find new rules that may help earlier detection of diseases. Such new rules or modified parameters can be deployed to the real-time analysis framework seamlessly. As can be seen in Figure 1, four distinct processes can be identified in the research edition framework.

1. *Data Ingestion:* A process that makes sure that RE stores all relevant data in the Hadoop-based platform.
2. *Data Enrichment:* Historical context that is generated from the data analytics component to bootstrap analytics and enrich incoming data on real-time processing component; more specifically, patient medical data or other related persistent data to enrich the live physiological data during the online processing.
3. *Adaptive Analytics:* Models that are generated by analytics such as data mining, machine learning, or statistical modeling in Hadoop platform used as basis for analytics on incoming physiological data in the real-time component and updated based on online observations.
4. *Data Visualization:* A process that visualizes data and information for different types of users.

In the “Sepsis Case Study” section, we elaborate the data flow and processing steps of the RE in which we describe one of our developed algorithms for detecting sepsis in neonates.

### Clinical Edition

**Figure 2:**
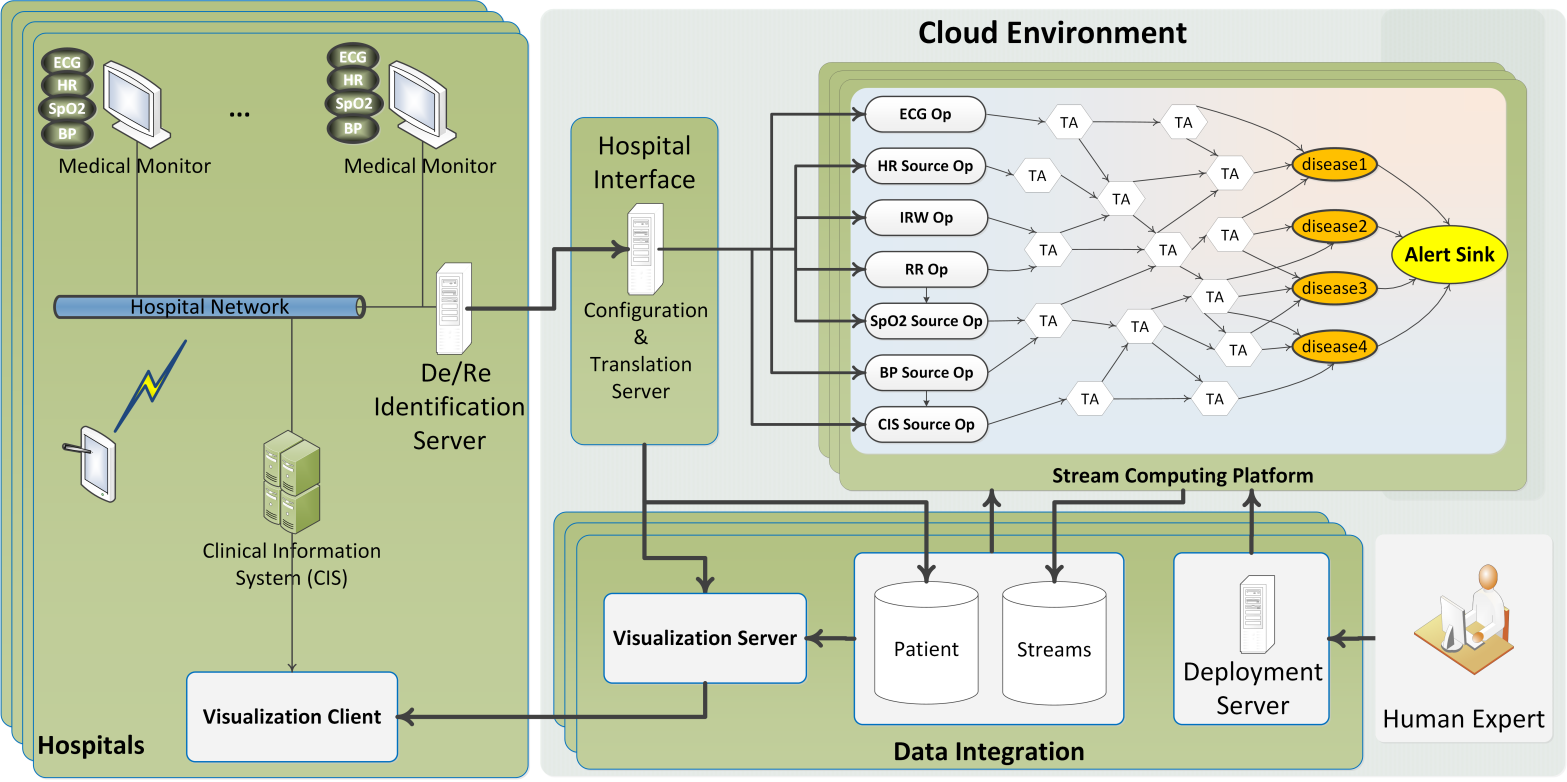
General architecture of the framework (clinical edition).

Clinicians, nurses, specialists, and other authorized hospital staff may use the clinical edition (CE; see Figure 2) to monitor their patients in a much more effective manner in real time. The CE can be considered as a CDSS that can continuously monitor a large number of patients simultaneously and automatically. This edition is capable of monitoring large numbers of patients’ physiological/clinical data and producing appropriate alarms in case of any medical complication onset. In addition, it can visualize a specific patient’s data either live or historically back a week or more. The ontology for the collection of high-speed synchronous physiological data provides a standardized terminology for acquired physiological data, including measurement metrics, sampling frequency, and acceptable ranges for the received values [19]. As with the collection of physiological data, asynchronous clinical data collection is supported by an ontology that specifies acceptable ranges for the collected values. Examples of clinical data include age, gender, medical history, and laboratory results. The core of the CE is a stream computing middleware component, which provides scalable processing of multiple streams of high-volume, high-rate data.

### High-Level Security and Privacy Schema

**Figure 3:**
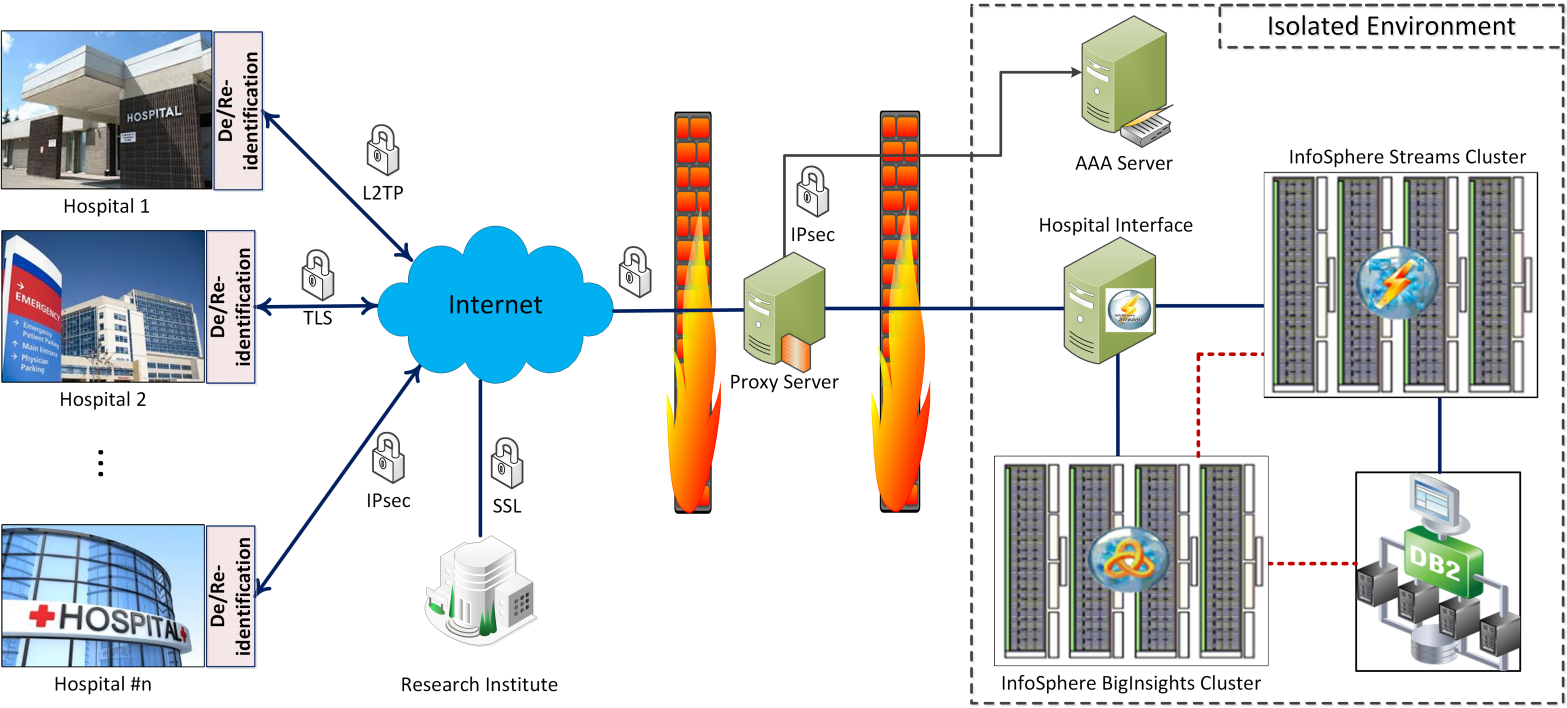
Security and privacy perspective of the Artemis-IC framework.

In this section, we present a high-level security architectural view of the framework. The details and implementation could vary depending on circumstances and applications. As can be seen in Figure 3, hospitals and research institutes are connected to the framework back end through secure channels. Two firewalls have been designed to isolate the framework from the outside world sequentially. The outer one separates the proxy server (ie, framework gateway), which is the edge server of the framework from the Internet. The inner firewall isolates the core of framework from the proxy server. Depending on the granularity of health analytics services, different type of users with various permission and data access levels could be defined. In Artemis-IC, we used a deidentification technique by which we eliminate the properties that might be used to identify patients. Personal data such as medical record number (MRN), name, address, and exact birth date were removed. The MRN was replaced with a study identifier with the translation between the two known only within the hospital. The exact date of birth was replaced with an admission age range of the form 0-3 days old, 4-7 days old, 8-10 days old, and greater than 10 days old. These ranges were chosen for clinically significant reasons. This process is performed in the De/Reidentification Server at hospitals (Figure 2).

### Tailoring of the Method for Monitoring Premature Babies

Premature birth, also known as “preterm birth,” is defined as birth before 37 weeks’ gestational age. It has been identified as one of the most important perinatal health problems in industrialized nations. NICUs internationally provide critical care for premature and ill term infants. Premature infants in NICUs can be as young as 23 weeks’ gestation [20].

Vital organ monitoring together with ventilation support and nutrition or drug titration through smart infusion pumps all generate large volumes of data at high frequency. An electrocardiogram (ECG) graph can be generated based on 1000 readings a second. Heart rate, respiration rate, and blood oxygen are displayed each second resulting in 86,400 readings each day. A premature newborn infant’s heart beats more than 7000 times an hour, which is approximately 170,000 times a day. Yet traditional charting protocols, whether documented on paper or within an EHR, typically enable the persistent storage of one value per hour of an indicative heart rate for that hour. A newborn infant’s neurological function could also be monitored resulting in multiple waveforms each generating tens of millions of data points per patient per day. Drug and nutrition infusion data from smart infusion pumps can be more than 60 different fields provided every 10 seconds. Given that these infants can have more than 10 infusions concurrently, infusion can generate more than 1 GB of drug infusion data from a single patient per day [21].

**Figure 4:**
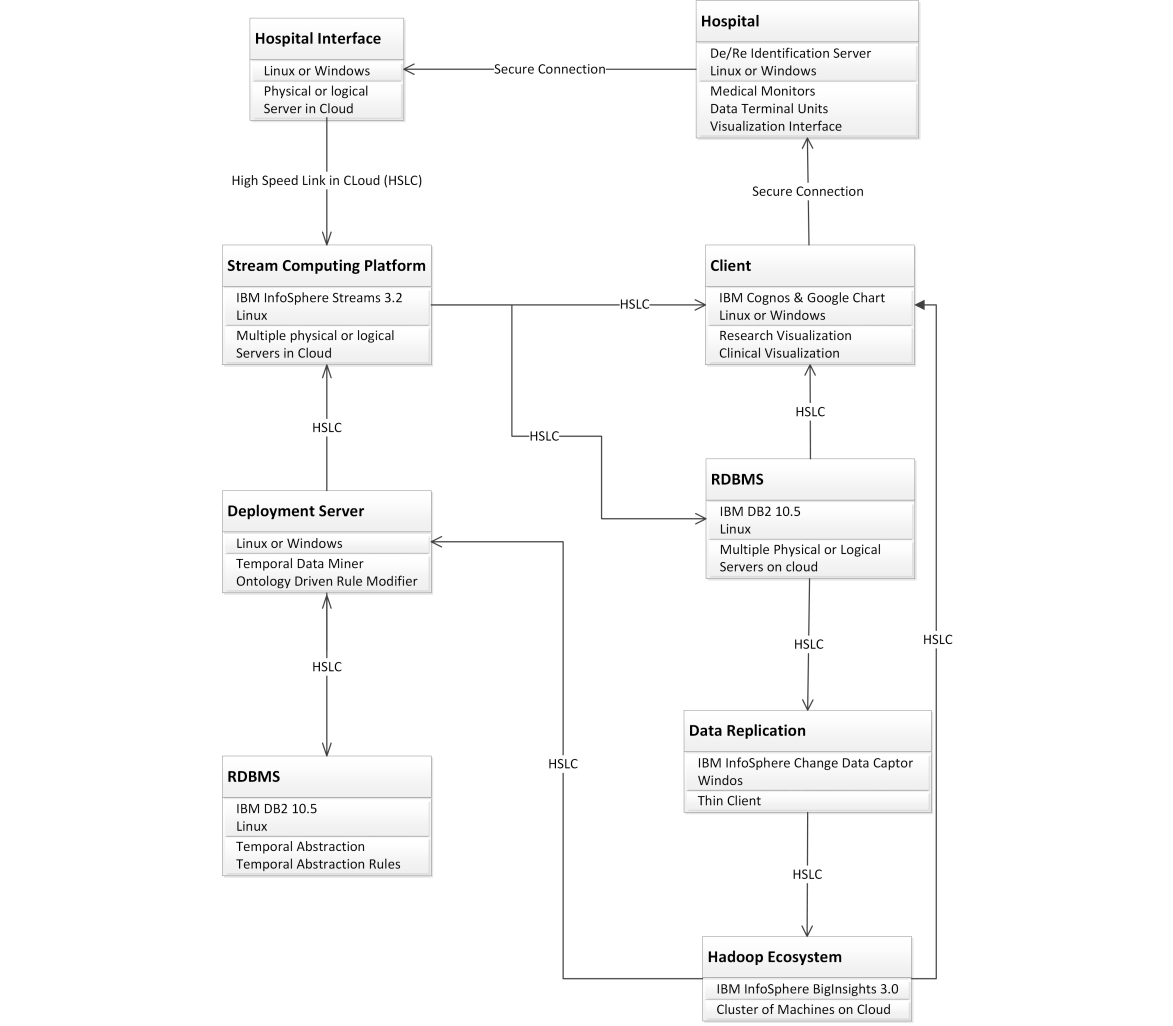
Artemis deployment at SickKids Hospital.

We propose a customized version of the framework, Artemis-IC, for monitoring preterm/surgical babies at NICUs. The Artemis-IC provides HAaaS for concurrent multipatient, multistream, and multidiagnosis through temporal analysis to support real-time clinical decision support and clinical research [22,23]. We deployed a pilot project by implementing Artemis-IC at Toronto’s SickKids hospital and proposed an analytical model [24] to enable performance evaluation and capacity planning in advance of final deployment. In addition, there is another pilot of the Artemis-IC at Women and Infants Hospital of Rhode Island (WIHRI), which is collecting physiological data for analytical and simulation modeling purposes. Figure 4 shows the customization and tools that we employed to deploy Artemis-IC framework in SickKids Hospital. As IBM is one of the partners in this research, we used IBM products to implement the framework.

To date, these environments (ie, SickKids and WIHRI deployments) support clinical studies on late-onset neonatal sepsis [22,25]; apnea of prematurity, in which the infant experiences pauses in breathing and reductions in heart rate and blood oxygen saturation [26]; retinopathy of prematurity, which can result in permanent blindness [27]; and pain [28].

Clinicians and researchers are leading these studies from different institutes toward the certification and formal approval of the medical algorithms. Algorithms for the Artemis-IC platform are developed either using data mining techniques that have not previously been detectable, such as our work on late-onset neonatal sepsis [22,25] or identifying patterns described in the medical literature using automated methods such as our work on apnea of prematurity [26]. These algorithms are validated in robust clinical trials before being used to provide decision support for clinicians. For example, the clinical rule states that “If a pause in breathing occurs for greater than 20 seconds, or a pause in breathing that is associated with a change in heart rate, or blood oxygen saturations happens,” then a reportable condition of apnea is present [26].

The current Artemis-IC implementations at SickKids and WIHRI have no impact on bedside care, as yet. We are comparing analytical results with current clinical observation and treatment practices to discover new patterns in real-time physiological data that could lead to the earlier detection and prevention of various diseases [26]. From first quarter 2015, we plan to deploy new research where we will be able to compare the results of using Artemis-IC with clinical outcomes using current clinical practices. Some of the algorithms that we have validated when they were running in parallel are due to be certified in 2015/2016 and will be deployed in target clinical institutions. We plan to provide experimental evaluation from multiple deployments of the Artemis-IC in our future reports.

### Sepsis Case Study

**Figure 5:**
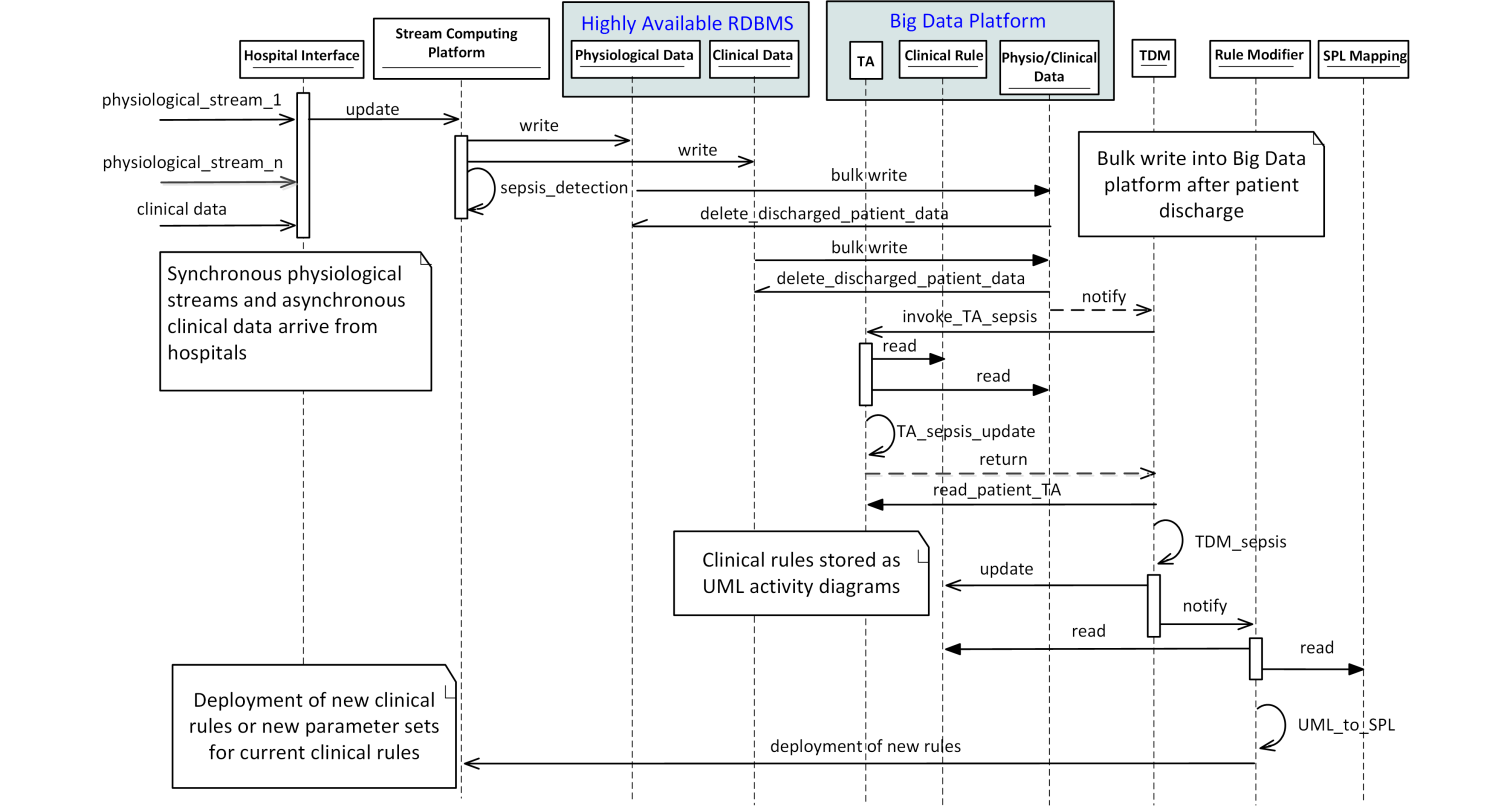
UML sequence diagram of sepsis detection and temporal data mining steps.

In this section, we elaborate the interactions between the main components of Artemis-IC for sepsis detection. Sepsis is a potentially life-threatening complication of an infection, which causes whole-body inflammation. In addition to real-time detection, we also demonstrate the knowledge extraction process in detail. The Unified Modeling Language (UML) sequence diagram shown in Figure 5 illustrates all steps including data acquisition, online detection, temporary data storage, persistent data storage within the big data platform, knowledge discovery, knowledge translation, and rule deployment.

Initially, multiple concurrent physiological data streams along with related clinical data are received by the hospital interface. Data are sent to the physiological and clinical database via the stream-computing platform. At the same time, the stream-computing platform runs the current deployed medical rule for sepsis detection. Upon patient discharge, their data including physiological and clinical data will be loaded into the big data platform by the relational database management system (ie, bulk move). Temporal abstractions (TAs) are then performed for the specific service of critical care, in this case sepsis detection, which involves (1) reading from the clinical rules and physiological/clinical tables, and (2) writing the patient TA to the TA table. Temporal data mining then can be performed on the TA results, possibly resulting in updates to the clinical rule table, after null hypothesis-based testing or other rule assessment, for example. Note that the resulting clinical rules are modeled in a UML concurrent activity diagram [19]. The rule modifier is notified of a rule modification and translates the UML representations of the new clinical rule to stream processing language (SPL) based on the SPL mappings active ontology. Finally, the new rule can be deployed on the stream-computing platform for upcoming real-time analysis. Note that the rule deployment on the Artemis-IC clinical edition will be performed under supervision of domain human experts as opposed to here where we consider the Artemis-IC research edition.

### Quality of Service

As the framework has a service-oriented architecture (SOA), the quality of service (QoS) is of great importance. To assign the proper amount of resources to each hospital, we present a method to create an analytical model to enable an accurate estimation of storage, memory, and computation power for the real-time health analytics components and retrospective analytics components. The model utilizes realistic patient population distribution that is based on gestation age characteristics and condition onset probabilities within those contexts. Both of these variables dictate the predicted length of stay for that infant. In the following section, we present the model within the context of SickKids hospital. In future work, we will do this for other hospitals before deployment. We also leave performance modeling of the research edition as our future work in which we concentrate on another type of users of the framework (ie, researchers).

### Analytical Modeling of the Method

**Figure 6:**
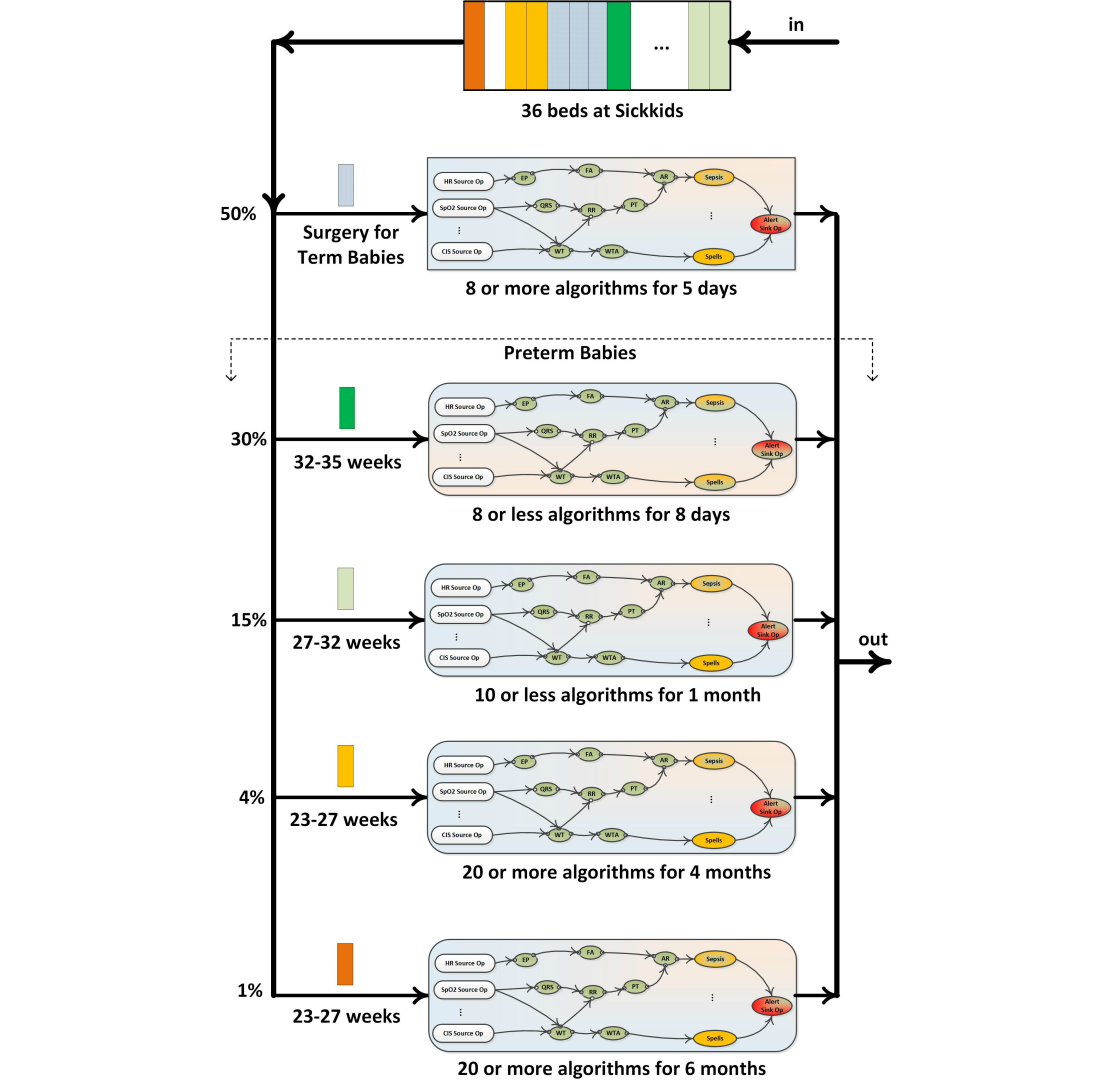
Types of patients and their medical service path at SickKids NICU.

The analytical modeling of Artemis-IC deployment at SickKids hospital’s NICU is required before any deployment because critical care units (CCUs)/ICUs are different in terms of types of patients, arrival process of patients, mean hospitalization time, type of services, required QoS, etc. Figure 6 shows the patient journey in the NICU at SickKids hospital. SickKids has 36 NICU beds including different types of patients. Depending on the type of patients, different numbers of algorithms for various periods will be triggered.

After discharging of a patient, a new patient will be submitted to NICU in 4-6 hours. Fifty percent of patients are term babies who are referred to SickKids for surgical purposes. Surgical babies stay in hospital for 5 days approximately, and 8 medical algorithms will be applied for after-surgery monitoring. The rest of patients, that is, preterm babies, are classified into three categories: babies who are born at 32-35, 27-32, and 23-27 weeks of their gestation age. The first group (ie, 30% of the patients) will be monitored by at most 8 medical algorithms for a mean period of 8 days. The second group (15%) of preterm babies will be monitored by 10 or fewer algorithms for an average time of 1 month. The third group is divided into two subclasses depending on medical conditions: 80% of this group (ie, 4% of the whole population) needs to be monitored by 20 or more algorithms for 4 months, and 20% (ie, 1% of the whole population) needs to be monitored by 20 or more algorithms for approximately 6 months. As Figure 6 suggests, SickKids NICU can be modeled as a single heterogeneous finite queue with multiple service facilities. Each type of patient has distinct characteristics in terms of length of stay and number of algorithms. Algorithms are also different in terms of required computational resources.

The SickKids NICU receives more admission requests than it has space for and prioritizes neonatal surgical patients. Other patients are typically redirected to either Sunnybrook Hospitals or Mount Sinai Hospital’s NICU when SickKids is operating at or near capacity. The total number of bed spaces available for admission is thus 118, with 40 and 42 of these spaces available at these other 2 hospitals, respectively. We model the Artemis-IC platform as an M/G/m/m queuing system (*M* stands for Markovian, ie, Poisson), which indicates that the interarrival time of patient’s arrival is exponentially distributed with the mean value of λ while patients’ resident time at NICU is independently and identically distributed random variables that follows a general distribution. The system under consideration contains *m* servers (ie, bed spaces) that renders service in order of patients’ arrivals (first-in-first-serve [FIFS]). The capacity of system is *m*, which means there is no extra room for queuing patients. As the population size of newborns is relatively high while the probability that a given newborn baby to be preterm is relatively small, the arrival process can be modeled as a Poisson process. The details of the performance modeling can be found in [24].

## Results

The analytical model has been implemented in Maple 17 [29] in order to obtain the numerical results. First, we characterize the performance metrics for the current configuration of Artemis-IC at SickKids that was described earlier in the section. Table 1 shows the performance metrics and important exogenous parameters. The average length of stay for patients is 16 days, and each patient requires 9 algorithms on average on the stream computing platform (ie, IBM Streams). The mean number of monitored patients (ie, occupancy rate) is 34.9, so that 311 algorithms will be running on Streams. Each algorithm is consuming approximately 110 MB of memory, which indicates the requirement of at least 32 GB of memory for the stream-computing cluster. Note that this amount of memory is just for application hosts and the management hosts require at least 2 GB more of memory.

**Table 1:** Configuration parameters and performance metrics for current capacity of SickKids NICU.

Parameter	Value
Beds in NICU, n	36
Patient arrival (patient/day), mean rate	4.5
Length of stay for patients (days), mean	16
Number of algorithms for 1 patient, mean	9
All running algorithms on Streams, n	311
NICU’s service (patient/day), rate	0.062
Blocking probability	0.455
Number of patients in NICU, mean	34.9
Memory per algorithm, mean MB	110
Required memory on Streams cluster, GB	32
Required CPU cores for Streams cluster, n	16
Required storage for a patient’s data (per day), MB	700
Required storage on BigInsights cluster (per year), TB	8.6

As can be seen in Table 1, the amount of minimum storage for the Hadoop cluster (ie, BigInsights cluster) to only support the accommodation of raw physiological data for 1 year is 8.6 TB. Depending on the data schema design on the BigInsights cluster, additional storage might be required for the metadata. Moreover, the storage required for nonphysiological data such as patient information, laboratory results, and other related medical data should be added on top of this calculation.

**Figure 7:**
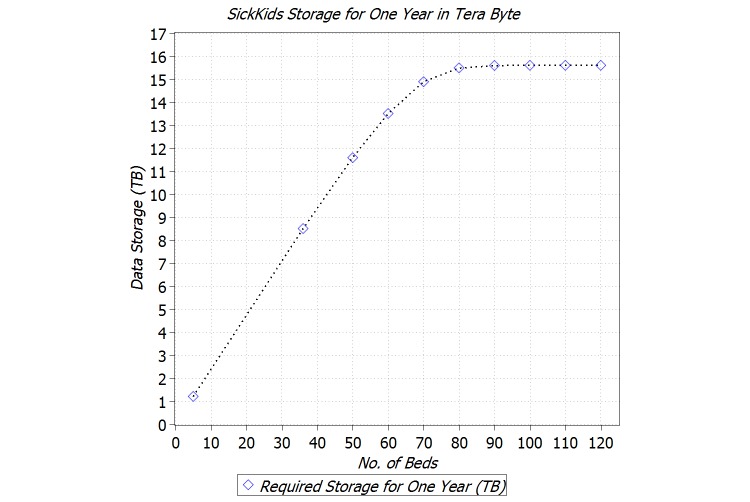
Required storage for BigInsights cluster for different conﬁgurations.

Figure 7 shows the amount of storage for the BigInsights cluster, for 10, 36, 50, 60, 70-120 beds in the NICU. Note that this amount is only for raw physiological data acquired from NICU. The amount of storage increases linearly with respect to NICU capacity up to 60 beds. Then between 60 and 80 beds, it is increases sublinearly and in the end flattens. After reaching the capacity of 90 beds, the amount of required storage remains unchanged, which indicates that the NICU entered into the unsaturated regime and can accommodate all new patient arrivals. In other words, for 1 year, 16 TB of storage is sufficient for the SickKids NICU regardless of NICU’s capacity (ie, the number of bed spaces).

**Figure 8:**
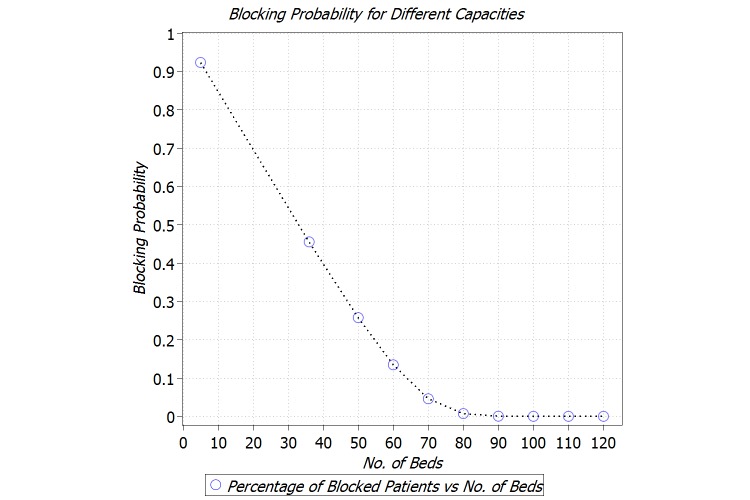
Blocking probability for different conﬁgurations.

We are also interested in studying the number of patients who get blocked, that is, redirected to another NICU, due to the capacity limitations of the NICU of interest. To this end, we characterize the blocking probability for the NICU with the capacity of 10-120 beds. As can be seen in Figure 8, for the current capacity of SickKids NICU (ie, 36 beds), 46% of patients get blocked. However, by increasing the capacity to 150 beds, the blocking will be less than 1%.

**Figure 9:**
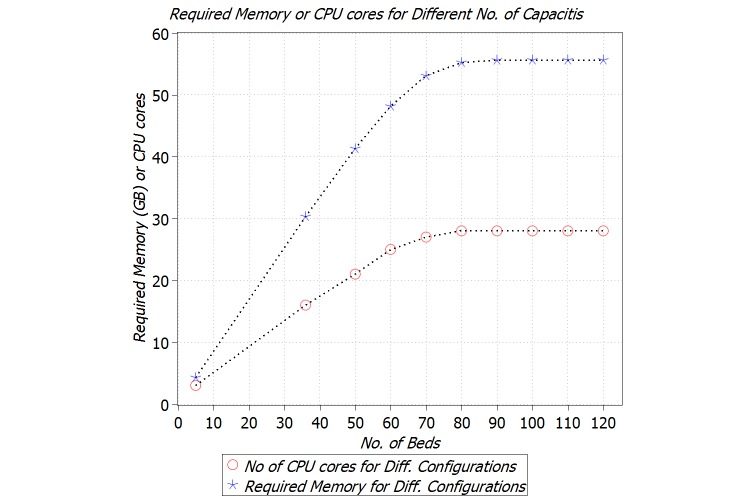
Required memory and computation power for Streams cluster for different conﬁgurations.

We also investigated the amount of memory and computation power for the stream-computing cluster for different configurations. Figure 9 shows the trend of required memory and number of CPU cores with respect to number of beds. For up to 70 beds, there is a linear dependency between the required memory and capacity; however, results show 60 GB of memory suffices for the Streams cluster based on these arrival and departure rates.

Our calculation for computation power is based on the standard CPU cores, that is, 2.00-GHz core, on IBM Softlayer cloud-based servers [30] and our experiments, which revealed that for each 20 algorithms we need a dedicated CPU core. The trend for computation power is almost similar to memory, explained above. We shall repeat the fact that these amounts of memory and computation power are just for application hosts. Depending on the deployment of management servers, extra resources might be needed.

## Discussion

### Principal Considerations

We have described and evaluated the design, implementation, and pilot deployments of a framework that provides health analytics as services. This framework can be considered as a general architecture that can be tailored for different use cases in the health informatics domain. One such customization is the Artemis-IC project that provides a way for clinicians to have online, real-time execution of the clinical rules in an intensive care environment. Moreover, Artemis-IC provides researchers with a rich set of easy access data and analytics tools by which knowledge discovery will be much more attainable than in the past. Because Artemis-IC’s target environments are critical care units, we have carried out extensive performance evaluation in order to guarantee expected quality of service and a high level of availability in particular. This work has three main aspects to be compared with similar works in the area, namely, data collection, real-time, and retrospective analysis. In the following sections, we compare our research to related work with regard to these three aspects.

### Data Collection

Collection of the physiological data is the first step in the development of a CDSS. As technology has progressed, the amount of physiological data as well as clinical information about patients, for example, EHR, has grown significantly [31]. As such, developing systems that record these data securely and at a suitable sampling rate and make them highly available is a research topic on its own [26,32,33].

Sukuvaara et al [34] developed a system called *DataLog*, which would connect to bedside monitors through an RS232 serial interface to collect physiological signals every 5 seconds. They performed some trending analysis on the signals and combined it with heuristic “if-then” rules to create a knowledge-based alarm system. However, capturing a data point once every 5 seconds is not enough to implement complex algorithms in the real-time environment, which is a part of our solution. In addition, only numeric signals are collected with DataLog, and no waveform data are captured, which is an important component of detecting conditions in real time.

Moody et al [35] developed customized software to log the signals coming from the Hewlett Packard content management system (Merlin) bedside monitors that were being used in the medical, surgical, and cardiac ICUs of Beth Israel Hospital, Boston, using a pair of RS232 serial interface cards in the monitor and communicating the data to a standard personal computer over a serial interface. They were able to record 3 ECG signals each sampled at 500 Hz and 4 or 5 other signals sampled at 125 Hz, in addition to periodic measurements and alarm messages. While the amount of data collected is impressive, their approach was to strictly record and store the data for the purpose of retrospective analysis. There was no functionality to serve the data for any online processing.

Saeed et al [36] designed a system that collected physiological and clinical data from the information management system on the hospital’s local area network for creating a temporal ICU patient database called *MIMIC II*. They monitored patients admitted to an 8-bed medical ICU and an 8-bed coronary care unit. The physiological data consisted of 4 continuously monitored waveforms (2 leads of ECG, arterial blood pressure, pulmonary artery pressure) sampled at 125 Hz, 1-minute parameters (heart rate, blood pressure, oxygen saturation, and cardiac output), as well as monitor-generated alarms. The strength in their approach is the ability to vary the presentation of data depending on the specific type of research for which the data are being used. Users of the database can extract a detailed record of a single signal, or more temporal analysis data from many signals can be displayed in one view. However, this ability to provide data temporally can be done only after considerable preprocessing and data fusion and is inherently retrospective.

A pilot and customized implementation of our method (ie, Artemis-IC) in SickKids Hospital is capable of collecting 15 data streams including 12 scalars (reading 1 integer per second) and 3 waveform streams (reading 60 doubles per second) and ECG (reading 512 double per second). In addition, the Artemis-IC clinical information system (CIS) adapter interfaces with the clinical information management system (CIMS) to access the SickKids CIMS patient EHR and stream the data into the framework [22].

### Real-Time Patient Monitoring

Current cutting-edge health informatics research projects aim to discover new condition onset behaviors that are evident in physiological data streams earlier than traditional detection of conditions in critical care data [23]. To this end, some hospitals may participate in pilot programs that aim to collect real-time patient data from network-enabled monitoring devices. These collected data are then analyzed to extract relevant temporal behaviors and usually stored for future data mining and analysis operations.

Historically, physiological stream monitoring of ICU patients has been provided by “black box” regulatory body-approved medical devices located at the patients’ bedside. While there has been a growing body of biomedical engineering and clinical research over the past 20-30 years proposing newer approaches for advanced physiological stream monitoring, they still predominantly have a physiological stream, clinical condition, or patient-centric approach [37]. Zhang et al [38] have discussed the implementation of a Health Data Stream Analytics System called the “Anesthetics Data Analyzer,” which has been developed to provide anesthetists with the ability to monitor and query trends in physiological signals data, a kind of stream data from the health care domain.

The BioStream [39] research project was designed to support the continuous monitoring of heart information of a patient on top of a general-purpose stream processing software architecture. The ECG was the main signal of interest. The goal of the group was to develop the prototype and collaborate with a medical institution on a pilot study. A Drexel University research team set out to design a system that performed online continuous processing of an ICU patient’s data stream and data capture to perform offline analysis to develop new clinical hypotheses [40].

As we propose a programmable component for the real-time processing in our solution, it can be customized to track a vast variety of diseases simultaneously. This capability is in part because of a comprehensive data collection followed by efficient ETL techniques that we employed in the design and implementation process. Moreover, there exist five active studies for developing and certifying medical algorithms to be deployed on the real-time component.

### Retrospective Analysis and Knowledge Discovery

The taxonomy for analytic workflow systems has already been presented [41]. Based on the taxonomy and a study of the existing analytic software and systems, the authors proposed the conceptual architecture of CLoud-based Analytics-as-a-Service (CLAaaS). They outline the features that are important for CLAaaS as a service provisioning system such as user- and domain-specific customization and assistance, collaboration, modular architecture for scalable deployment, and service level agreement (SLA). We considered the aforementioned outlined features for designing the proposed framework in this work.

Analytics have been utilized in various aspects of health care including predictive risk assessment, clinical decision support, home health monitoring, finance, and resource allocation [6]. The proliferation of big data and analytics in health care has spawned a growing demand for clinical informatics professionals who can bridge the gap between the medical and information sciences.

John Tukey pioneered the use of exploratory data analysis nearly four decades ago [42]. Various packages and languages that support exploratory data analysis have been developed since. This includes S, S-Plus, R, SPSS, SAS, OLAP, and MATLAB [43,44]. A recent view of modern data exploration practices is available from Behrens and Yu [45]. All these approaches can be used as the knowledge discovery engine in our proposed architecture.

The retrospective analysis of previously persistently stored physiological data through the determination and assessment of TA-based qualitative behaviors from the analysis of quantitative physiological data has been widely employed. However, research is either physiological stream-clinical condition or patient centric [1]. A structured approach for the translation of the knowledge gained from this research, which is predominantly statistical and sometimes more recently data mining in nature, has been lacking [37,46].

One approach to the Software-as-a-Service utilizes the SOA approach to software design where software services are made available to the cloud through a series of Web services. Examples of early work showing the potential for the use of cloud computing in health care are emerging [11,47]; however, these research efforts do not provide functional support to critical care. McGregor [48,49] proposes a functional set of Web services to support critical care as part of her solution manager service as applied to health care. However, aspects such as rule definition are not clearly defined within that functional set. The application of cloud computing for the provision of a service of critical care supporting both real-time patient monitoring and retrospective clinical research remains an open research problem.

### Strengths and Limitations

One of the main strengths of our work is the openness of the proposed framework. It is general enough to be applied to various scenarios in health informatics. The stream computing platform in the clinical edition can be programmed for monitoring different types of patients including but not limited to neonates, children, adults, and the elderly in critical care units, home, work, and even in transit. Any medical diagnostic approach that can be described algorithmically can be deployed and programmed on a real-time processing unit. Another key strength of the framework is the modular design of the architecture. In the research edition, any interested big data solution can be utilized. For example, any Hadoop distribution (eg, Cloudera [50], Hortonworks [51]) or other big data analytics tools such as Spark [52] can be employed for different types of retrospective analytics, provided that different types of analytics such as machine learning, statistical modeling, batch processing, interactive, streaming, graph, and in-memory analysis are accessible to researchers. In addition, our experience in customization of the framework for the NICU revealed that it could be deployed with minimum intervention with current procedures and policies. For example, for Artemis-IC deployment at SickKids we used only the spare port at the bedside monitors. We also developed an interface to interact with the clinical management information system to get the EHR from the hospital. Moreover, the systematic performance modeling can be easily extended or customized to support other medical care units. Estimation and prediction of the appropriate underlying infrastructure is no longer an unknown question.

However, there exist some limitations that need to be addressed properly and according to the target deployment. First and foremost is adopting appropriate privacy mechanisms for the physiological and medical data. For Artemis-IC, we used a simple deidentification technique that might not be completely secure and efficient. We use this technique to enable a simple reidentification process at hospitals. A more robust approach may apply encryption and perform analytics on encrypted data [53]. A second challenge is the ETL process for physiological data. This process should eliminate noise inputs from valid data efficiently; this is a research topic on its own [54,55]. Third, the process of medical algorithms certification is a complex and time-consuming process that prevents acquiring actual benefits out of the system in a timely manner. In other words, the lack of standardization seems to be an obstacle toward the adoption of systems such as Artemis-IC.

### Conclusion

Our work fills the gap by providing a solution that can utilize the latest achievements in cloud-based analytics for health care informatics; it provides both real-time and retrospective analysis capabilities for various stakeholders. Moreover, we proposed a performance model that can be used for the capacity planning of the Artemis-IC in advance of its physical deployment. Artemis-IC and the corresponding performance model can be tailored for other ICUs as well; the architecture is plug-in–based so that similar open-source or commercial components can be integrated to realize the solution. Artemis-IC can also be deployed on any other cloud environment (ie, cloud agnostic).

## Abbreviations

Artemis-IC: Artemis In Cloud
CDSS: clinical decision support system
CE: clinical edition
CIMS: Clinical Information Management System
CLAaaS: CLoud-based Analytic-as-a-Service
EHR: electronic health record
ETL: extract, transform, load
HAaaS: Health-Analytic-as-a-Service
ICU: intensive care unit
NICU: neonatal intensive care unit
QoS: quality of service
RE: research edition
SLA: service level agreement
SOA: service-oriented architecture
SPL: Stream Processing Language
SSL: secure socket layer
TA: temporal abstraction
UML: Unified Modeling Language
WIHRI: Women and Infants Hospital of Rhode Island
